# Supplementary material for: Genetic Dissection of Grain Yield and Agronomic Traits in Maize under Optimum and Low-Nitrogen Stressed Environments
Source: Int J Mol Sci. 2020 Jan 15;21(2):543. doi: 10.3390/ijms21020543 (PMC7013417; doi:10.3390/ijms21020543)
Supplement: Supplementary file 1 [file ijms-21-00543-s001.pdf]

Supplementary Table1. Genetic characteristics of detected QTL for anthesis date (AD) under optimum, low nitrogen stress in main season (LNM) and off-season (LNO) in DH lines derived from five bi-parental populations.

Population	Mgt	Chr	Pos (cM)	LeftMarker	RightMarker	LOD	PVE(%)	TPVE(%)	Add	Fav Allele
CML550/CML494	OPT	3	227	S3_152451120	S3_155619613	4.46	5.46	71.31	0.13	CML494
	OPT	3	355	S3_1289855	S3_171466703	3.22	3.88		-0.11	CML550
	OPT	4	100	S4_228926221	S4_228626317	13.47	20.21		0.26	CML494
	OPT	5	218	S5_196031436	S5_206019269	8.69	11.81		-0.20	CML550
	OPT	6	26	S6_162568586	S6_161010798	4.58	5.90		0.14	CML494
	OPT	7	152	S7_174157338	S7_173807263	9.21	12.27		-0.20	CML550
	OPT	8	245	S8_151911852	S8_152261359	10.64	14.96		0.22	CML494
	OPT	8	267	S8_142233374	S8_137468517	15.30	27.79		-0.30	CML550
	LNM	2	347	S2_31924520	S2_34925673	3.45	11.32	28.86	-0.24	CML550
	LNM	3	265	S3_45035564	S3_47832327	3.27	10.28		0.23	CML494
	LNO	1	426	S1_198541547	S1_198279139	3.11	7.25	47.27	-0.30	CML550
	LNO	1	519	S1_27140851	S1_26197963	4.42	10.51		-0.36	CML550
	LNO	4	131	S4_183134905	S4_181871673	3.71	8.68		0.33	CML494
	LNO	7	146	S7_175566913	S7_174157338	5.64	14.26		-0.41	CML550
CML550/CML504	OPT	1	43	S1_282409602	S1_283186611	3.73	4.47	46.88	-0.25	CML550
	OPT	1	292	S1_69865657	S1_69288842	5.40	6.44		-0.30	CML550
	OPT	3	67	S3_200876966	S3_201584853	4.18	4.90		0.27	CML504
	OPT	4	61	S4_224308438	S4_224048022	3.13	3.67		0.23	CML504
	OPT	5	457	S5_45438168	S5_44985543	30.55	50.60		-0.84	CML550
	OPT	5	466	S5_41538958	S5_40652438	18.05	26.80		0.62	CML504
	OPT	8	21	S8_168815355	S8_168493048	3.17	3.67		-0.23	CML550
	OPT	8	107	S8_135070884	S8_130930928	34.71	57.59		-1.05	CML550
	LNM	1	42	S1_284504632	S1_282409602	56.25	95.81	58.04	1.14	CML504
	LNM	1	383	S1_17383245	S1_14803778	10.23	10.49		-0.37	CML550
	LNM	2	229	S2_183919141	S2_184646201	7.38	7.05		-0.32	CML550
	LNM	6	240	S6_162558564	S6_168794605	5.44	5.07		0.26	CML504
	LNM	7	271	S7_112877861	S7_110568982	4.03	3.79		-0.22	CML550
	LNM	10	3	S10_150087021	S10_146943516	3.41	3.19		0.21	CML504
	LNO	1	351	S1_32079580	S1_33832111	34.66	81.62	33.58	1.00	CML504
	LNO	9	147	S9_104435623	S9_102698508	4.60	7.36		-0.30	CML550
CML550/CML511	OPT	1	343	S1_52345244	S1_230179861	8.16	29.42	29.02	-2.99	CML550
	LNM	1	343	S1_52345244	S1_230179861	3.18	12.75	12.04	-2.23	CML550
	LNO	1	343	S1_52345244	S1_230179861	12.52	39.42	46.11	-3.56	CML550
	LNO	1	521	S1_46413710	S1_42476919	4.60	12.06		-0.39	CML550
CML505/LaPostaSeqC7-F64-2-6-2-2-B-B	OPT	4	264	S4_69843767	S4_67493486	4.93	13.41	13.36	0.31	CML505
	LNM	1	771	S1_66013917	S1_60755570	3.21	9.11	8.00	0.29	CML505
	LNO	1	392	S1_220785207	S1_221241110	4.64	10.49	25.45	-0.39	LP
	LNO	5	445	S5_421	S5_141945888	5.31	15.99		0.40	CML505
CML536/LaPostaSeqC7-F64-2-6-2-2-B-B	LNM	9	141	S9_112940495	S9_111715623	8.42	26.35	37.71	0.44	CML536
	LNM	10	59	S10_89984330	S10_90815324	3.29	9.19		0.26	CML536
	LNO	1	415	S1_49415609	S1_48379091	4.27	13.51	29.69	-0.36	LP
	LNO	8	136	S8_147917080	S8_148274279	3.22	9.95		-0.30	LP

Supplementary Table 2. Genetic characteristics of detected QTL for anthesis silking interval (ASI) under optimum, low nitrogen stress in main season (LNM) and off-season (LNO) in DH lines derived from five bi-parental populations.

Population	Mgt	Chr.	Pos (cM)	Left Marker	Right Marker	LOD	PVE(%)	TPVE(%)	Add	Fav Allele
CML550/CML504	OPT	3	77	S3_204126924	S3_206481369	4.91	7.58	31.26	0.06	CML504
	OPT	5	376	S5_169668014	S5_163945834	3.43	5.32		-0.05	CML550
	OPT	7	263	S7_119597893	S7_113205468	3.70	5.59		-0.05	CML550
	OPT	9	122	S9_119779555	S9_119132452	3.08	4.67		-0.05	CML550
	LNM	1	300	S1_66387567	S1_65350627	4.12	7.04	24.10	-0.09	CML550
	LNM	3	49	S3_197718647	S3_196434589	6.13	11.54		0.12	CML504
	LNM	10	180	S10_3908652	S10_1148472	3.76	7.16		-0.09	CML550
	LNO	8	97	S8_139630981	S8_135070884	3.61	7.52	12.84	0.10	CML504
CML505/LaPostaSeqC7-F64-2-6-2-2-B-B	OPT	3	200	S3_183867892	S3_199561708	4.57	12.94	11.70	0.17	CML505
	LNO	3	205	S3_193795900	S3_186485761	3.40	8.29	19.68	0.10	CML505
	LNO	3	357	S3_151334181	S3_149229159	4.04	9.79		-0.12	LP
CML536/LaPostaSeqC7-F64-2-6-2-2-B-B	LNO	1	466	S1_27505154	S1_26435510	8.84	27.12	39.41	-0.53	LP
	LNO	3	163	S3_213298747	S3_211719240	3.66	9.98		-0.32	LP

Supplementary Table 3. Genetic characteristics of detected QTL for plant height (PH) under optimum, low nitrogen stress in main season (LNM) and off-season (LNO) in DH lines derived from five bi-parental populations.

Population	Mgt	Chr	Pos (cM)	Left Marker	Right Marker	LOD	PVE(%)	TPVE(%)	Add	Fav Allele
CML550/CML494	LNM	3	224	S3_46511540	S3_153262861	3.70	14.00	20.40	2.04	CML550
	LNO	3	179	S3_172906641	S3_168838491	3.83	12.85	26.45	1.08	CML494
	LNO	9	62	S9_151147419	S9_150224858	4.71	17.13		1.23	CML494
CML550/CML504	OPT	1	46	S1_283186611	S1_280222332	8.32	8.04		-1.74	CML550
	OPT	1	107	S1_237562292	S1_236572842	3.80	3.39		-1.13	CML550
	OPT	1	206	S1_195754378	S1_194942819	15.72	15.89		-2.44	CML550
	OPT	2	141	S2_219659850	S2_218462880	3.64	3.20	59.82	1.09	CML504
	OPT	3	53	S3_197718647	S3_196434589	4.18	3.80		1.22	CML504
	OPT	8	341	S8_75951924	S8_77725407	10.78	10.40		1.98	CML504
	OPT	9	224	S9_21192733	S9_19527579	3.75	3.33		-1.26	CML550
	LNM	1	46	S1_283186611	S1_280222332	7.69	7.23		-2.14	CML550
	LNM	1	206	S1_195754378	S1_194942819	17.30	16.96		-3.27	CML550
	LNM	1	271	S1_86945521	S1_83434475	6.44	5.60	61.72	-1.88	CML550
	LNM	3	82	S3_206195841	S3_208333232	3.77	3.19		1.46	CML504
	LNM	7	231	S7_126750234	S7_125835674	4.30	3.66		-1.52	CML550
	LNM	8	334	S8_92199584	S8_94575375	14.50	13.92		2.99	CML504
	LNO	1	48	S1_283186611	S1_280222332	8.08	10.25	49.52	-1.27	CML550
	LNO	1	206	S1_195754378	S1_194942819	12.49	16.56		-1.62	CML550
	LNO	8	301	S8_136912486	S8_137757371	3.94	4.71		0.87	CML504
CML550/CML511	LNM	1	343	S1_52345244	S1_230179861	5.52	14.26	44.52	-8.12	CML550
	LNM	6	127	S6_96152977	S6_94700288	3.37	8.30		-1.20	CML550
	LNM	8	65	S8_145140385	S8_137303469	4.53	11.71		1.44	CML550
	LNO	1	343	S1_52345244	S1_230179861	3.78	13.77	24.33	-8.34	CML550
CML505/LaPostaSeqC7-F64-2-6-2-2-B-B	LNO	3	190	S3_194824065	S3_195397829	3.04	8.48	8.05	-1.54	LP
CML536/LaPostaSeqC7-F64-2-6-2-2-B-B	LNO	8	156	S8_162185699	S8_166372615	3.58	14.03	13.39	1.79	LP

Supplementary Table 4. Genetic characteristics of detected QTL for ear height (EH) under optimum, low nitrogen stress in main season (LNM) and off-season (LNO) in DH lines derived from five bi-parental populations.

Population	Mgt	Chr	Pos (cM)	LeftMarker	RightMarker	LOD	PVE(%)	TPVE(%)	Add	Fav Allele
CML550/CML494	LNM	3	84	S3_207741357	S3_206481439	3.05	6.16	53.16	-1.02	CML550
	LNM	3	219	S3_46177572	S3_46511540	8.26	19.32		1.83	CML494
	LNM	5	177	S5_188667809	S5_191088426	5.69	12.14		-1.64	CML550
	LNM	7	300	S7_17479195	S7_17180908	5.91	12.87		-1.53	CML550
	LNM	8	267	S8_142233374	S8_137468517	7.89	20.00		-1.84	CML550
	LNO	8	268	S8_130729873	S8_123153386	4.40	15.68	24.29	-1.12	CML550
CML550/CML504	OPT	1	25	S1_292891535	S1_291386678	5.45	5.50	56.64	-1.07	CML550
	OPT	1	183	S1_219232023	S1_217114738	11.92	12.43		-1.61	CML550
	OPT	1	292	S1_69865657	S1_69288842	10.46	10.67		-1.49	CML550
	OPT	3	66	S3_200596344	S3_200876966	4.16	4.07		0.94	CML504
	OPT	6	222	S6_153666432	S6_154619899	3.60	3.42		0.85	CML504
	OPT	10	9	S10_146943516	S10_144137339	6.44	7.00		1.22	CML504
	OPT	10	157	S10_6373041	S10_5482369	3.76	3.65		-0.87	CML550
	LNM	1	136	S1_230879834	S1_230186447	3.80	3.98	52.26	-1.14	CML550
	LNM	1	206	S1_195754378	S1_194942819	4.12	4.34		-1.17	CML550
	LNM	3	82	S3_206195841	S3_208333232	3.61	3.79		1.12	CML504
	LNM	4	200	S4_71535960	S4_62855411	7.57	10.05		-1.79	CML550
	LNM	8	329	S8_101755334	S8_95745623	4.66	5.08		1.27	CML504
	LNM	10	110	S10_73624067	S10_34023384	3.03	3.15		-1.00	CML550
	LNO	1	46	S1_283186611	S1_280222332	3.48	6.27	27.42	-0.55	CML550
	LNO	1	206	S1_195754378	S1_194942819	6.86	12.59		-0.78	CML550
	LNO	1	392	S1_15735866	S1_14031653	5.27	9.40		-0.67	CML550
CML550/CML511	OPT	1	343	S1_52345244	S1_230179861	4.65	16.71	22.87	-12.84	CML550
	OPT	1	514	S1_50951450	S1_49392612	3.15	11.15		-2.06	CML550
	LNM	1	343	S1_52345244	S1_230179861	4.72	16.79	23.70	-7.82	CML550
	LNM	1	513	S1_51752437	S1_50951450	3.35	11.51		-1.28	CML550
CML505/LaPostaSeqC7-F64-2-6-2-2-B-B	OPT	5	168	S5_179858396	S5_177665119	3.72	9.94	14.79	0.89	CML505
	LNM	5	397	S5_11374553	S5_9616695	3.59	9.20		0.68	CML505
CML536/LaPostaSeqC7-F64-2-6-2-2-B-B	LNM	7	32	S7_166270114	S7_163512547	3.71	13.01	23.10	-1.38	LP
	LNO	8	156	S8_162185699	S8_166372615	3.05	12.11	11.40	0.93	CML536

Supplementary Table 5. Genetic characteristics of detected QTL for ear position (EPO) under optimum, low nitrogen stress in main season (LNM) and off-season (LNO) in DH lines derived from five bi-parental populations.

Population	Mgt.	Chr.	Pos (cM)	LeftMarker	RightMarker	LOD	PVE(%)	TPVE(%)	Add	Fav Allele
CML550/CML494	OPT	6	10	S6_166674013	S6_163629442	3.04	13.14	10.53	0.00	CML494
	LNM	8	262	S8_148261703	S8_148007217	5.25	18.69	23.30	-0.01	CML550
	LNO	8	263	S8_147097779	S8_146474892	8.04	28.90	28.75	-0.01	CML550
CML550/CML504	OPT	1	311	S1_58363752	S1_57288598	8.90	12.81	38.35	0.00	CML504
	OPT	3	110	S3_221158754	S3_221901376	4.23	5.77		0.00	CML504
	OPT	4	84	S4_191754231	S4_191538363	3.85	5.32		0.00	CML504
	OPT	4	246	S4_36724590	S4_33663643	6.86	9.80		0.00	CML504
	OPT	8	72	S8_152113114	S8_151194215	6.04	9.61		0.00	CML504
	LNM	1	311	S1_58363752	S1_57288598	7.78	12.03	33.54	0.00	CML504
	LNM	4	57	S4_227110696	S4_226284987	3.62	5.36		0.00	CML504
	LNM	4	248	S4_34335173	S4_34552572	7.30	11.25		0.00	CML504
	LNO	1	298	S1_67649374	S1_66013609	3.75	5.50	38.34	0.00	CML504
	LNO	1	392	S1_15735866	S1_14031653	3.26	4.70		0.00	CML504
	LNO	4	82	S4_193745467	S4_193147210	3.60	5.22		0.00	CML504
	LNO	6	158	S6_112817455	S6_114440133	3.13	4.47		0.00	CML504
	LNO	8	80	S8_151194215	S8_149801553	5.13	7.54		0.00	CML504
CML550/CML511	OPT	1	621	S1_10362814	S1_6174894	4.36	15.14	28.05	-0.01	CML550
	OPT	2	310	S2_15909091	S2_13288169	3.94	12.85		0.01	CML511
CML505/LaPostaSeqC7-F64-2-6-2-2-B-B	OPT	5	124	S5_186676927	S5_185412709	3.87	9.94	15.30	0.00	CML505
CML536/LaPostaSeqC7-F64-2-6-2-2-B-B	LNO	1	51	S1_271764010	S1_273696896	4.15	12.86	31.04	0.01	CML536
	LNO	1	400	S1_55503537	S1_53413566	5.21	16.55		0.01	CML536

Supplementary Table 6. Genetic characteristics of detected QTL for senescence (SEN) under optimum, low nitrogen stress in main season (LNM) and off-season (LNO) in DH lines derived from five bi-parental populations.

Population	MGT	Chr.	Pos (cM)	Left Marker	Right Marker	LOD	PVE(%)	TPVE(%)	Add	Fav allele
CML550/CML504	OPT	1	168	S2_203937216	S2_204466878	4.42	7.62	23.65	0.03	CML504
	OPT	3	404	S3_134682595	S3_137089605	3.66	6.34		0.02	CML504
	OPT	6	249	S6_162558564	S6_168794605	3.35	6.15		-0.02	CML550
	LNM	1	204	S1_198219595	S1_197055941	4.21	8.12	15.06	-0.01	CML550
	LNM	2	174	S2_202138908	S2_201146790	3.44	6.51		0.01	CML504
	LNO	3	68	S3_200876966	S3_201584853	6.38	8.57	45.87	-0.06	CML550
	LNO	3	537	S3_181558923	S3_177129159	3.66	4.88		0.04	CML504
	LNO	5	309	S5_201939197	S5_85666905	4.65	8.77		0.08	CML504
	LNO	6	187	S6_131027488	S6_131760613	22.66	35.66		0.11	CML504
	LNO	9	37	S9_153413533	S9_149896475	4.83	6.14		0.05	CML504
